# Effectiveness of advanced nursing care on depression in patients with ovarian cancer: A protocol of systematic review of randomized controlled trial

**DOI:** 10.1097/MD.0000000000015316

**Published:** 2019-04-26

**Authors:** Zhen-hua Lu, Xiao-qin Kang

**Affiliations:** Department of Gynecology and Obstetrics, The Second Affiliated Hospital of Shaanxi University of Chinese Medicine, Xianyang, China.

**Keywords:** advanced nursing care, depression, effectiveness, ovarian cancer, randomized controlled trial

## Abstract

**Background:**

This systematic review will assess the effectiveness of advanced nursing care (ANC) on depression in patients with ovarian cancer (OC).

**Methods:**

We will identify any relevant randomized controlled trial from Cochrane Library, MEDLINE, Embase, Web of Science, Springer, Chinese Biomedical Literature Database, and China National Knowledge Infrastructure from their inceptions to March 5, 2019. The primary outcome includes depression. The secondary outcomes consist of anxiety, quality of life, and adverse events. Data that meets all the eligibility criteria will be extracted, pooled, and analyzed by using RevMan 5.3 software. Methodological quality for each eligible study will be assessed by using Cochrane risk of bias tool.

**Results:**

This study will analyze depression, anxiety, quality of life, and adverse events of ANC on depression in patients with OC.

**Conclusion:**

The findings of this study will provide the latest evidence for the effectiveness and adverse events of ANC on depression in patients with OC.

**Ethics and dissemination:**

No ethic approval is required for this study, because all the data will be extracted from previous published studies. The results of this study will be presented at conference or will be published at a peer-reviewed journal.

**PROSPERO registration number:**

PROSPERO CRD42019126374.

## 1 Introduction

Ovarian cancer (OC) is one of the most leading causes of death among female population, with a 5-year survival rate of ∼ 47%.^[1–3]^ Although early diagnosis can help improve survival, only 15% of OC are diagnosed at early stage.^[4]^ It has been estimated that about 22,240 new cases of OC will be reported in 2018 and 14,080 associated mortalities reported in the United States in 2017.^[5–7]^

Many patients with OC often have psychological disorders, such as depression and anxiety, especially for depression.^[8–11]^ Previous studies have reported that patients with OC with depression are significantly more risk of higher mortality rates, and poorer treatment outcomes.^[12,13]^ The prevalence of depression across all cancer patients varies from 14% to 56%.^[14–16]^ Thus, the effective managements for OC patients with depression are very important and necessary.

Lots of studies have reported that advanced nursing care (ANC) is effective for OC patients with depression.^[17–24]^ However, no systematic review has investigated its effectiveness for OC patients with depression. Therefore, in this study, we will assess the effectiveness of ANC for the OC patients with depression systematically.

## 2 Methods

### 2.1 Eligibility criteria for included studies

#### 2.1.1 Study type

We will collect studies of randomized controlled trials (RCTs) that assessed the effectiveness of ANC on depression in patients with OC. However, we will not consider studies of nonclinical studies, noncontrolled studies, and non-RCTs.

#### 2.1.2 Participants

All patients with OC are clinically diagnosed depression disorder will be included in this study, without restrictions of country, race, gender, etc.

#### 2.1.3 Interventions

All patients in the experimental group receive ANC only for depression therapy. Any other therapies will not be considered, including the combination of ANC with other interventions. In addition to the ANC, all patients in the control group undergo any treatments.

#### 2.1.4 Outcomes

Primary outcome is depression. It can be measured by any relevant scales, such as Hamilton Depression Rating Scale.

Secondary outcomes are anxiety (as assessed by Hamilton Anxiety Rating Scale or others), quality of life (as evaluated by 36-Item Short Form Health Survey or any others); and any adverse events.

### 2.2 Literature search strategy

We will identify any potential studies from Cochrane Library, MEDLINE, Embase, Web of Science, Springer, Chinese Biomedical Literature Database, and China National Knowledge Infrastructure from their inceptions to March 5, 2019. In addition, we will also search websites of clinical registry, and reference lists of relevant studies. The detailed sample of search strategy for Cochrane Library is shown in Table 1. Similar search strategies for other databases will also be used.

**Table 1:**
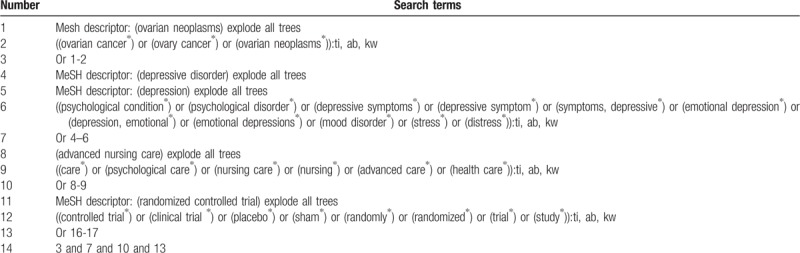
Search strategy utilized in Cochrane Library database.

### 2.3 Data collection

#### 2.3.1 Study selection

**Figure 1:**
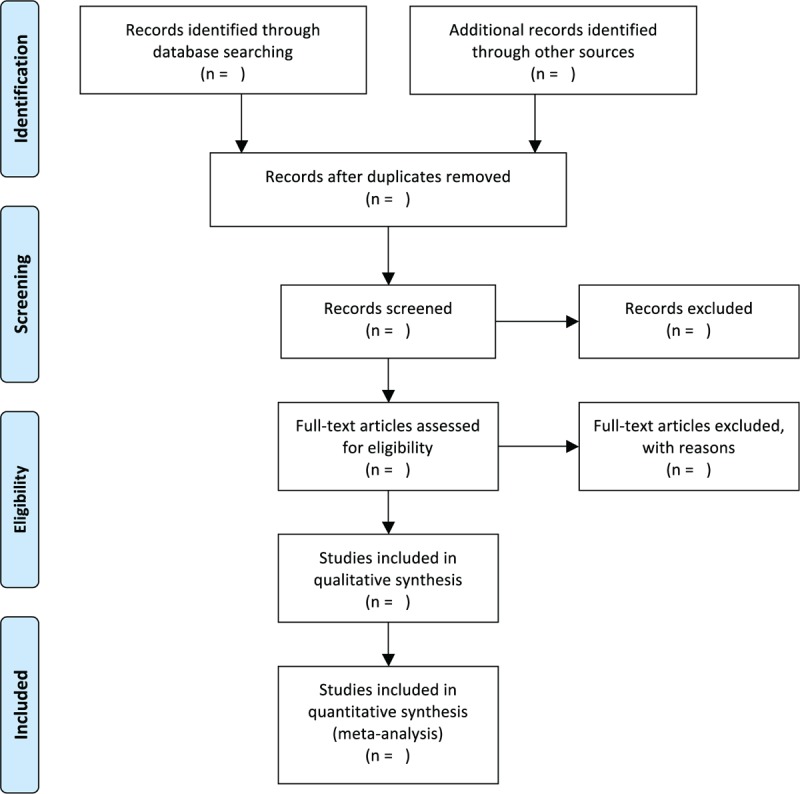
Flowchart of study selection.

Two experienced authors will guide the literature search, and NoteExpress 3.2.0 will be utilized to manage the retrieved results. Both of them will independently scan the title and abstract for each record, and exclude irrelevant studies. Then, full texts will be read to further identify potential studies according to the predefined inclusion criteria. Any disagreements regarding the study selection will be solved by discussion with a third experienced author. The selection process is presented in Figure 1.

#### 2.3.2 Data extraction

Two experienced authors will extract data based on the standard data extraction form. The collected information include study characteristics (such as title, first author's name, country, published year, etc.), patient characteristics (such as sample size, diagnostic criteria, gender, etc.), study methods (such as randomization, blinding, etc.), details of treatment, and clinical outcome measurements. Divergences will be solved by a third experienced author through discussion.

#### 2.3.3 Missing data management

When any data are missing or insufficient, we will contact primary study authors by using email. When those missing data are not achievable, we will only analyze the available data, and discuss its impact as a limitation.

### 2.4 Risk of bias assessment for included studies

Two experienced authors will assess the risk of bias for each eligible trial by using Cochrane Risk of Bias Tool independently. This tool comprises of 7 items, and each item is further divided as 3 different levels: high, unclear, or low risk of bias. A third experienced author will help to solve any divisions through discussion.

### 2.5 Data synthesis and analysis

We will utilize RevMan 5.3 software to pool the data and carry out the data analysis. Continuous data will be presented as mean difference or standardized mean difference with 95% confidence intervals (CIs). Dichotomous data will be recorded as risk ratio with 95% CIs. *I*^*2*^ test will be used to investigate the heterogeneity, and it will be interpreted as follows: *I*^*2*^ ≤50% is considered as low level of heterogeneity; *I*^*2*^ > 50% indicated high level of heterogeneity. A low heterogeneity suggests little variability among eligibility studies, and data will be pooled by using a fixed-effect model, and meta-analysis will be conducted if it is possible. When high heterogeneity occurs among the eligible trials, a random-effect model will be used to pool the data, and analyze the data. Meanwhile, subgroup analysis will be carried out to identify any possible reasons that may cause high heterogeneity. When there is still high heterogeneity after subgroup analysis, we will not pool the data, and outcome results will be reported as narrative summary.

### 2.6 Additional analysis

Subgroup analysis will be conducted according to the different intervention types, study quality, location and treatment duration. Sensitivity analysis will be carried out to determine the robustness of pooled results by removing low quality studies.

### 2.7 Reporting bias

Reporting bias will be assessed by using funnel plot^[25]^ and Egg's regression^[26]^ when more than 10 eligible trials are included in this study.

## 3 Discussion

OC is one of the most common gynecologic cancers in female patients. Most patients with OC also suffer from depression disorder. Although several managements can help relieve depression in patients with OC, it is not always effective for some patients. Several previous clinical trials have reported that ANC can help to manage the depression for patients with OC effectively. However, up to presently, no study has systematically assessed its effectiveness for patients with OC.

This study is the first study to systematically evaluate the effectiveness of ANC for depression in patients with OC. The findings of this study will summarize latest evidence of the ANC for managing depression in patients with OC. It will also inform our understanding of ANC for depression in patients with OC across all previous published clinical trials.

## Author contributions

**Conceptualization:** Xiao-qin Kang.

**Data curation:** Zhen-hua Lu, Xiao-qin Kang.

**Funding acquisition:** Zhen-hua Lu.

**Investigation:** Xiao-qin Kang.

**Methodology:** Zhen-hua Lu, Xiao-qin Kang.

**Project administration:** Xiao-qin Kang.

**Resources:** Zhen-hua Lu, Xiao-qin Kang.

**Software:** Zhen-hua Lu.

**Supervision:** Xiao-qin Kang.

**Validation:** Zhen-hua Lu, Xiao-qin Kang.

**Visualization:** Zhen-hua Lu, Xiao-qin Kang.

**Writing – original draft:** Zhen-hua Lu, Xiao-qin Kang.

**Writing – review & editing:** Zhen-hua Lu, Xiao-qin Kang.
